# Supplementary material for: Multiplex Label-Free Kinetic Characterization of Antibodies for Rapid Sensitive Cardiac Troponin I Detection Based on Functionalized Magnetic Nanotags
Source: Int J Mol Sci. 2022 Apr 19;23(9):4474. doi: 10.3390/ijms23094474 (PMC9102693; doi:10.3390/ijms23094474)
Supplement: Supplementary file 1 [file ijms-23-04474-s001.zip › ijms-1673082-supplementary.pdf]

Supplementary Materials

Multiplex Label-Free Kinetic Characterization of Antibodies for Rapid Sensitive Cardiac Troponin I Detection Based on Functionalized Magnetic Nanotags

Alexey V. Orlov [1,*], Juri A. Malkerov [1,2], Denis O. Novichikhin [1,2], Sergey L. Znoyko [1] and Petr I. Nikitin [1,2,*]

[1] Prokhorov General Physics Institute of the Russian Academy of Sciences, 38 Vavilov St, 119991 Moscow, Russia; jurimalkerov@yandex.ru (J.A.M.); nammen@yandex.ru (D.O.N.); znoykos@yandex.ru (S.L.Z.);

[2] National Research Nuclear University MEPhI (Moscow Engineering Physics Institute), 31 Kashirskoe Shosse, 115409, Moscow, Russia

* Correspondence: alexey.orlov@kapella.gpi.ru (A.V.O.); petr.nikitin@nsc.gpi.ru (P.I.N.)

S1. Optimization of rapid sensitive assay for cTnI detection based on magnetic nanotags

The assay conditions were optimized to maximize the ratio of the specific magnetic signal in the antigen presence (1 ng/mL of cTnI) to the non-specific magnetic signal under the antigen absence (0 ng/mL of cTnI). The following parameters were optimized: amount of capture antibody immobilized on the magnetic particles, amount of magnetic particles added to the analyzed sample, concentration of tracer antibody deposited on the test strip.

S.1.1. Optimization of the amount of capture antibody immobilized on the magnetic particles

The optimal amount of capture antibody immobilized on the magnetic particles was found to be 50 μg of antibody per 1 mg of the particles (Table S1).

Table S1. Comparison of the specific and non-specific magnetic signals measured at different amounts of capture antibody immobilized on the magnetic particles

Amount of capture antibody immobilized on the magnetic particles, μg per 1 mg of MP	Specific magnetic signal (1 ng/mL), a.u.	Non-specific magnetic signal (0 ng/mL), a.u.
6.25	38.4 ± 4.6	0.8 ± 0.6
12.5	63.1 ± 2.5	1.2 ± 0.5
25	98.7 ± 4.9	0.8 ± 0.7
50	174 ± 17	1.2 ± 0.5
100	232 ± 28	1.7 ± 0.8

S.1.2. Optimization of the amount of magnetic particles added to the analyzed sample

The optimal amount of the magnetic particles added to the analyzed sample was determined to be 5 μg (Table S2).

Table S2. Comparison of the specific and non-specific magnetic signals measured at different amounts of the magnetic particles added to the analyzed sample

Amount of magnetic particles added to the analyzed sample, μg	Specific magnetic signal (1 ng/mL), a.u.	Non-specific magnetic signal (0 ng/mL), a.u.
2.5	90.5 ± 6.7	0.9 ± 0.6
5	184 ± 14	1.5 ± 0.5
10	351 ± 22	4.2 ± 3.1
20	712 ± 66	8.1 ± 5.5

S.1.3. Optimization of the concentration of tracer antibody deposited on the test strip

The optimal concentration of tracer antibody deposited on the test strip was 2 mg/mL (Table S3). The concentrations exceeding 2 mg/mL were not studied for the judicious conservation of the reagents.

Table S3. Comparison of the specific and non-specific magnetic signals measured at different concentrations of the tracer antibody deposited on the test strip

Concentration of tracer antibody deposited on the test strip, mg/mL	Specific magnetic signal (1 ng/mL), a.u.	Non-specific magnetic signal (0 ng/mL), a.u.
0.5	67.9 ± 4.7	0.8 ± 0.5
1.0	110 ± 6.5	1.0 ± 0.7
2.0	199 ± 11	1.5 ± 1.1

S2. Investigation of the specificity of rapid sensitive assay for cTnI detection based on magnetic nanotags

The assay specificity was verified in the experiments with serum samples that contained non-target molecules instead of cTnI. The following non-target molecules, including other cardiomarkers, were tested: fatty-acid-binding cardiac protein (1000 ng/mL), pro-brain natriuretic peptide (1000 ng/mL), thyrotropic hormone (300 ME/mL), biotin (3000 ng/mL), and chloramphenicol (100 ng/mL). The absence of false-positive signals (Table S4) in the experiments with non-target molecules indicates the assay specificity.

Table S4. Magnetic signals recorded in the experiments with various non-target molecules

Analyte	Concentration	Magnetic signal, a.u.
cardiac troponin I	1 ng/mL	194 ± 8.7
fatty-acid-binding cardiac protein	1000 ng/mL	1.2 ± 0.5
pro-brain natriuretic peptide	1000 ng/mL	1.7 ± 1.1
thyrotropic hormone	300 ME/mL	1.3 ± 0.9
biotin	3000 ng/mL	1.9 ± 0.4
chloramphenicol	100 ng/mL	1.0 ± 0.8
